# Bioinspired electrospun janus nanofibrous dressing for synergistic antibacterial activity and tissue regeneration

**DOI:** 10.3389/fmicb.2026.1788110

**Published:** 2026-04-10

**Authors:** Jinying Song, Zhongfei Gao, Yicong Zhang, Ying Li, Yuqing Zhao, Huanhuan Yan, Xiaojing Jiang, Hongxia Zheng, Yuna Zhang, Anyan Yao, Chengbo Li, Guige Hou, Xianrui Xie, Meng Zhang

**Affiliations:** 1 Key Laboratory of Medical Antibacterial Materials of Shandong Province, School of Pharmacy, Binzhou Medical University, Yantai, China; 2 Shandong Junxiu Biotechnology Co., Ltd., Yantai, China

**Keywords:** antibacterial, collagen, electrospinning, quaternized polyurethane, wound healing

## Abstract

**Introduction:**

Wound infection remains significant challenge in clinical healing, frequently leading to delayed tissue regeneration and prolonged inflammatory responses.

**Methods:**

Herein, a bioinspired electrospun Janus nanofibrous dressing (QAS-PU/FC) was designed to mimic the hierarchical architecture of natural skin and achieve synergistic infection inhibition and tissue regeneration.

**Results:**

The dressing is characterized by an Janus structure, in which the outer layer, composed of quaternary ammonium acrylate-based polyurethane and polycaprolactone (PCL), provides robust antibacterial protection and mechanical integrity, while the inner layer, composed of collagen and PCL, offers a biocompatible matrix that supports cell adhesion, proliferation, and tissue remodeling. The Janus design creates a functionally graded interface, enabling sequential antibacterial defense and regenerative stimulation. The outer antibacterial surface effectively inhibits both *Staphylococcus aureus* and *Escherichia coli* colonization, while the inner collagen-rich layer promotes fibroblast migration and neovascularization. *In vivo* studies further demonstrated that the Janus dressing significantly suppressed bacterial infection, reduced inflammation, and accelerated wound closure, achieving nearly complete tissue regeneration with organized collagen deposition and re-epithelialization.

**Discussion:**

This work provides a promising bioinspired strategy to design multifunctional Janus electrospun membranes for infection-controlled and regenerative wound care applications.

## 1 Introduction

Chronic and infected wounds remain a significant clinical challenge worldwide, frequently leading to delayed tissue regeneration, prolonged inflammatory responses, and increased risk of subsequent complications (Chen et al., 2023; Gou et al., 2024). Effective wound management requires an integrated therapeutic strategy that can simultaneously prevent bacterial infection and promote tissue repair (Zhong et al., 2022). However, conventional wound dressings, such as gauze, hydrogels, or single-function polymeric films, generally fail to achieve the synergistic balance between antimicrobial defense and regenerative promotion (Rezvani Ghomi et al., 2023). These limitations highlight the urgent need for multifunctional wound dressings with both antibacterial and pro-healing properties (Boyko et al., 2022).

In recent years, electrospinning technology has emerged as a versatile platform for fabricating nanofibrous membranes with tunable structure, porosity, and composition (Doodmani et al., 2024). Electrospun nanofibers can mimic the architecture of native extracellular matrix (ECM), providing a biomimetic microenvironment that supports cell adhesion, migration, and proliferation (Pan et al., 2015; Qu et al., 2024; Tan et al., 2022). Furthermore, their large surface-to-volume ratio allows the incorporation of bioactive agents for antibacterial or pro-healing purposes (Li A. et al., 2022). Despite these advantages, most existing electrospun dressings exhibit homogeneous structures, which limit their ability to achieve spatiotemporal regulation of antibacterial activity and tissue regeneration.

The natural skin exhibits a distinct bilayer architecture consisting of the epidermis and dermis, each fulfilling unique physiological functions essential for wound protection and regeneration (Juncos Bombin et al., 2020; Clausen and Agner, 2016). The epidermis forms the primary physical barrier that prevents water loss and protects underlying tissues from pathogenic invasion. Its densely packed keratinocytes and lipid-rich intercellular matrix create an effective antimicrobial shield that limits bacterial colonization and penetration (Nguyen et al., 2020; Chieosilapatham et al., 2021). Building on the protective role of the epidermis, quaternary ammonium salts (QAS) represent a class of potent cationic antimicrobial agents widely utilized in biomedical materials (Leong et al., 2020; Obłąk et al., 2019). QAS exert bactericidal activity by electrostatically binding to the negatively charged bacterial cell membranes, leading to membrane destabilization, disruption of osmotic balance, and eventual cell lysis (Leong et al., 2020; Obłąk et al., 2019; Yu et al., 2025). Their contact-killing mechanism provides rapid and broad-spectrum antibacterial effects without relying on the slow diffusion of antibiotics, thereby reducing the risk of drug resistance (Yu et al., 2025; Zhou et al., 2023; Sun et al., 2025). When incorporated into polymer matrices, QAS can endow wound dressings with durable surface antibacterial functionality, helping maintain a sterile microenvironment during the early stages of wound healing (Aydin et al., 2019). The research by Iurilli et al. (2025), Goisis et al. (2025) focused primarily on “collagen-coated, silver nanoparticle-functionalised electrospun poly(lactic acid) (PCL) membranes.” The researchers employed uniform PCL fibre membranes that were coated with collagen and doped with silver nanoparticles (AgNPs). However, free silver ions may induce bacterial resistance and have limited antibacterial activity against *S. aureus*.

Beneath the epidermis, the dermis is rich in collagen, the major structural protein of the extracellular matrix (ECM), and plays a central role in orchestrating tissue repair following injury (Ricard-Blum, 2011; Schwarz et al., 2022). Collagen provides essential biochemical cues and a fibrillar scaffold that supports fibroblast adhesion, migration, and proliferation (Ricard-Blum, 2011). Additionally, collagen modulates inflammatory responses, promotes angiogenesis, and guides the deposition and remodeling of newly formed ECM. These properties make collagen indispensable for restoring tissue architecture and accelerating wound closure. Its ability to foster a microenvironment conducive to cell–matrix interactions is particularly critical in chronic or infected wounds where regenerative processes are impaired (Davison-Kotler et al., 2019).

**SCHEME 1:**
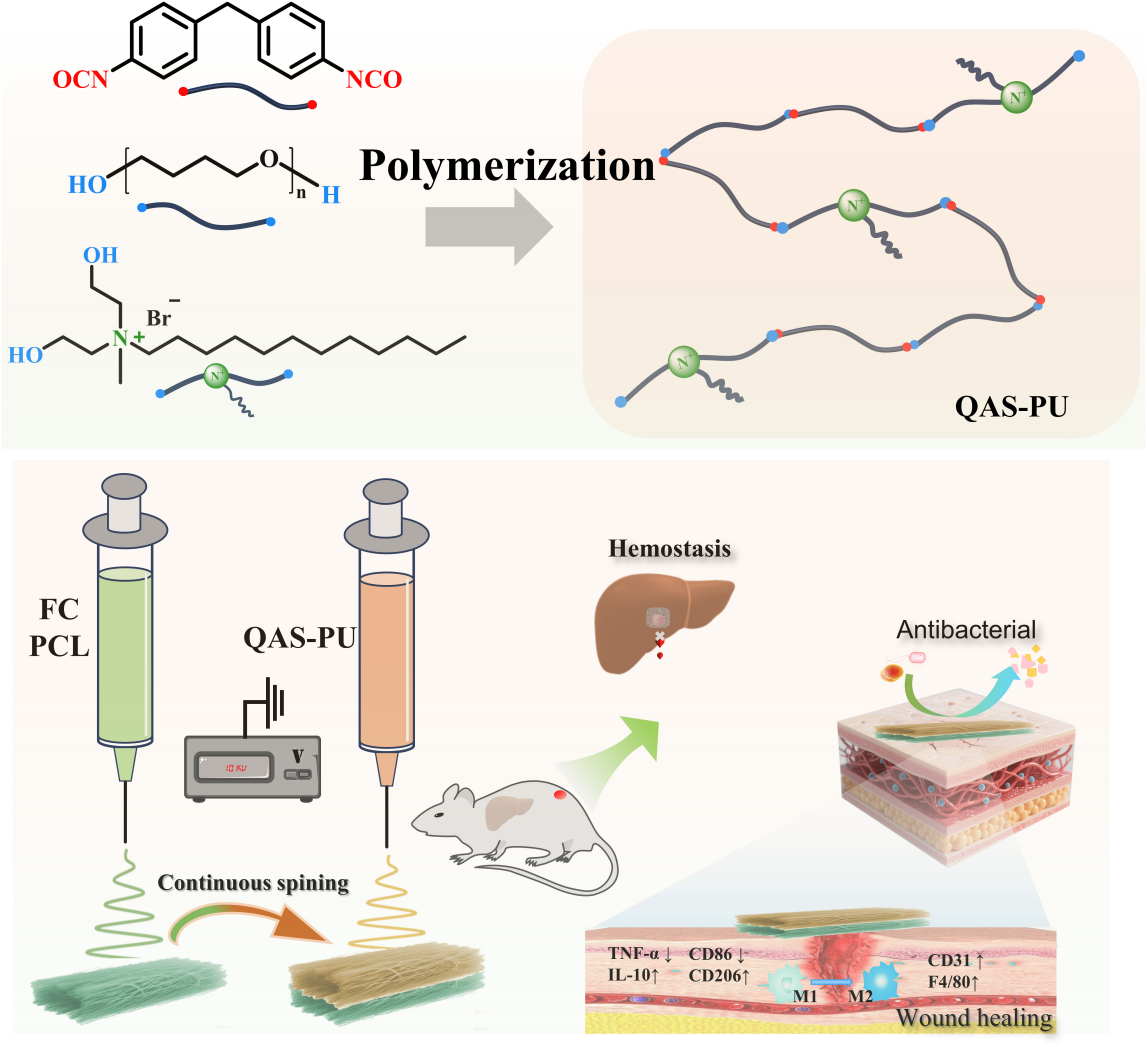
Schematic diagram of the preparation process and application of the QAS-PU/FC dual-layer fiber membrane in the healing of infected wounds.

Inspired by the hierarchical and asymmetric organization of natural skin, this study develops a Janus nanofiber dressing that functionally mimics the epidermis–dermis structure. The design integrates a QAS-modified polyurethane outer layer to provide robust antibacterial protection with a collagen/PCL inner layer engineered to support cell infiltration and tissue regeneration. This bioinspired Janus configuration aims to achieve sequential therapeutic functions, effectively addressing the dual challenges of infection control and tissue repair in complex wound environments (Scheme 1).

## 2 Materials and methods

### 2.1 Materials

Hexafluoroisopropanol (HFIP, purity = 99.5%), dodecyl dimethyl ethyl ammonium bromide (purity = 98%), Polytetramethylene ether glycol (Mn≈2,000) were supplied by Macklin, Ethylenediamine (EDA, 98%) was supplied by Rhawn, *N,N*-Dimethylformamide 99.8%, SuperDry, with molecular sieves, Water ≤ 50 ppm (by K.F.), 4,4’-Methylenediphenyldiisocyanate (4,4’-MDI). *Staphylococcus aureus* (ATCC27853) and *Escherichia coli* (ATCC25922) were purchased from American Type Culture Collection. Yeast powder and tryptone were obtained from the British OXOID Company, agar was purchased from Biofroxx GmbH, Germany, DMEM medium was purchased from Cytiva, USA, and trypsin *w*as purchased from Hyclon*e*, USA. *S. aureus* (ATCC6538) and *E. coli* (ATCC25922) were purchased from the Shanghai Biological Science Culture Collection Center. Unless otherwise specified, all other reagents were commercially available.

Scanning electron microscope (EVO LS15, Zeiss, Germany), Electronic universal tensile testing machine (CMT8502, China), Thermogravimetric simultaneous thermal analyzer (DSC3+/1100 LF, METTLER TOLEDO, Switzerland), Fourier transform infrared spectrometer (FT-IR, Nicolet IS50, USA), Contact angle analyzer (SZ-CAMD33, Shanghai Sunzern Instrument Co., Ltd., China), Electrospinning apparatus (YFSP-T, Tianjin Yunfan Technology Co., Ltd., China), Consumable-free high-throughput automatic cell counter [Model HD-4, Gaofen (Beijing) Biotechnology Co., Ltd., China].

### 2.2 Preparation of QAS-PU

In the pre-polymerization stage,4 g Polytetramethylene ether glycol (molecular weight ≈ 2,000) was weighed into a three-necked flask and 1.1 g 4,4’-M*e*thylenediphenyldiisocyanate was added. The mixture was stirred continuously and heated to 60 °C–70 °C, and this temperature was maintained for approximately 30 min. Subsequently, 10 mg N-methyl-N-dodecyl-N, N-bis(2-hydroxyethyl) ammonium bromide was added, and the reaction was allowed to proceed for an additional 10 min to obtain the prepolymer. In the chain-extension stage, the prepolymer was diluted to approximately 50% (w/v) with N, N-dimethylformamide, after which the temperature was increased to 70 °C. Ethylenediamine was then introduced as the chain extender and the mixture was allowed to react until a glycerol-like product was formed. During the termination step, 2-hydroxyethyl methacrylate was added as a grafting agent, followed by 21 μL of methyl methacrylate. After further reaction, a polyurethane grafted with quaternary ammonium and acrylate was obtained.

### 2.3 Preparation of QAS-PU/FC nanofibrous membranes

Initially, the surface polyurethane fiber membrane must be prepared, followed by the electrospinning of collagen. The Janus electrospun membranes were fabricated using an electrospinning apparatus. Preparation of FC electrospun fiber membranes: Dissolve the FC/PCL composite at an 8:2 weight ratio in a 1:1 volume ratio of HFIP and 10% acetic acid, stirring until dissolved. To prepare QAS-PU electrospun fiber membranes, polyurethane and polycaprolactone were dissolved in HFIP at a weight ratio of 5:2 and stirred at room temperature until dissolved. Detailed electrospinning parameters are provided in the Supplementary materials.

### 2.4 Characterization of QAS-PU and nanofibrous membranes

NMR test, the solvent was deuterated TFAA and the ^1^H NMR spectrum of QAS-PU was obtained; The FT-IR test was performed by setting the scanning wave number interval 4,000–500 cm^–1^, and the spectral results were processed by OMNIC software for graphical analysis.

Water absorption capacity, water contact angle, water vapor permeability, tensile property, thermal behavior, cytotoxicity assay, cell proliferation assay, cell scratch wound assay, antibacterial assay, *in vitro* hemostasis test and hemolytic properties are outlined in the Supplementary materials.

### 2.5 *In vivo* wound healing assay

BALB/c mice with a body mass of 25 ± 2 g were selected for the wound healing experiments. All procedures were performed with the approval of the Committee on the Ethics of Animal Experiments of Binzhou Medical University (protocol number: 2022–390). Detailed experimental procedures and the results of the immunohistochemical staining for TNF-α and IL-10 are included in the Supplementary materials.

### 2.6 Histology and immunofluorescence staining

Histology and immunofluorescence staining were performed to evaluate angiogenesis, collagen deposition and inflammatory cells during wound healing. F4/80, CD31 and CD86 were chosen for immunofluorescence staining. Detailed experimental procedures are provided in the Supplementary materials.

## 3 Results and discussion

### 3.1 Preparation and characterization of electro spun nanofibers

#### 3.1.1 FTIR spectra analysis

**FIGURE 1:**
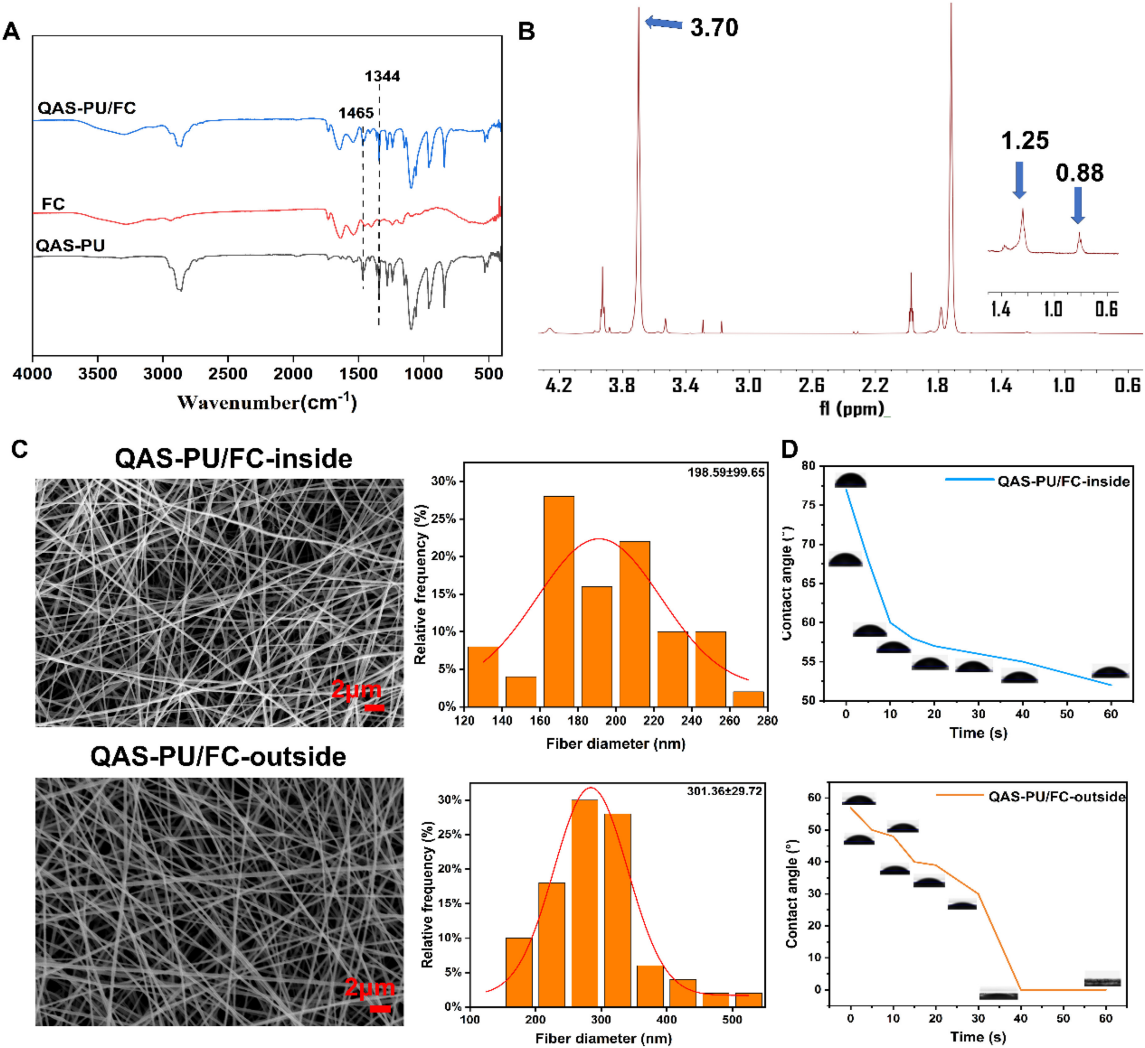
**(A)** The FTIR spectra of QAS-PU/FC, QAS-PU, and FC. **(B)** The 1H NMR spectrum of QAS-PU/FC. 29**(C)** SEM images and diameter distribution of QAS-PU/FC inner and outer layers. **(D)** Water contact angles of the inner and outer layers of janus nanofiber dressings.

The absorption peak at 1,465 cm^–1^ corresponds to the C-H bending vibration in the quaternary ammonium salt molecule. The peak observed at 1,344 cm^–1^ corresponds to the C-N stretching vibration present in the quaternary ammonium salt molecule (Figure 1A).

#### 3.1.2 Surface chemical structures

The peaks at 0.80 and 1.25 ppm correspond to the hydrogen nuclei signals of the methyl and methylene groups of the long-chain dodecane, which indicates the presence of long aliphatic chains. Further evidence of the successful grafting of quaternary ammonium salts onto the polyurethane chain was also detected. The peak at 3.70 ppm corresponds to the methoxy proton signal of methyl methacrylate. This suggests that methyl methacrylate has been successfully grafted onto the quaternary ammonium salt polyurethane (Figure 1B).

#### 3.1.3 Fiber morphology

The development of dressings that closely resemble the natural extracellular matrix (ECM) of natural skin tissue was of paramount importance for skin tissue engineering (Li T. et al., 2022). The images of the inner and outer layers of the double-layered nanofibers reveal a uniform morphology with non-directional orientation, and are free from bead-like or cracked structures (Figure 1C). The diameters of 50 nanofibers were measured from each image, with a range of 50 to 300 nm. However, the diameter of QAS-PU nanofibers was approximately 301.36 ± 29.72 nm. This increase in diameter may be attributed to the grafted acrylates enhancing the fibers’ tensile strength, thereby aligning their properties more closely with those of natural dermal collagen fibers, while concurrently enhancing their mechanical properties (Li T. et al., 2022; Parry et al., 1978).

#### 3.1.4 Contact angle analysis

The wettability of nanofibers has been demonstrated to play a crucial role in wound healing and scar inhibition, and water contact angle (WCA) measurement is an important method for evaluating wound surface wettability (Guo et al., 2020). The results (Figure 1D) indicate that all three fiber membranes exhibit hydrophilicity, with FC demonstrating the strongest hydrophilicity, while QAS-PU and QAS-PU/FC show comparable hydrophilic properties. The hydrophilicity of QAS-PU is attributable to the hybrid electrospinning of PEG and quaternized polyurethane, which imparts a degree of wettability to the fiber surface.

#### 3.1.5 Water vapor transmission rate and water uptake rate

**FIGURE 2:**
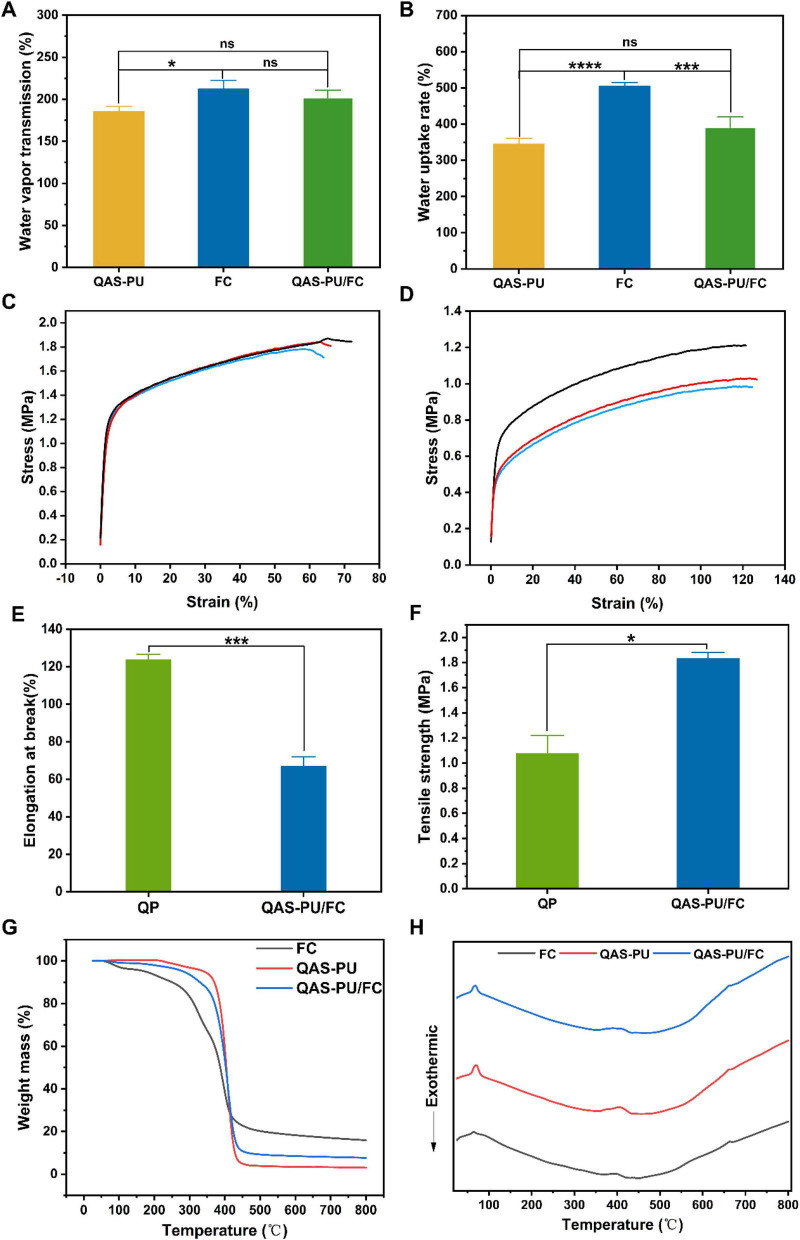
**(A)** Water vapor transmission rate (*n* = 3). **(B)** Water uptake rate (*n* = 3). **(C)** Stress-strain curve of QP (*n* = 3). **(D)** Stress-strain curve of QAS-PU/FC (*n* = 3). **(E)** Elongation at break (*n* = 3). **(F)** Tensile strength (*n* = 3). **(G)** TGA curve. **(H)** DSC curve. *p < 0.05, ***p < 0.001, ****p < 0.0001, ns means insignificant.

The water vapor transmission rate is indicative of a dressing’s capacity to enable gas exchange, thus preventing wound dehydration and excessive exudate accumulation. The primary function of a dressing is to absorb wound exudate, thereby drawing excess fluid away from the wound site while maintaining optimal moisture levels within the wound. The present study determined the water vapor transmission rate and water absorption capacity of three fiber membranes (Figures 2A, B). No significant differences were observed in water vapor transmission rates among the three nanofiber membranes, all of which exceeded 30%. Water absorption rates exceeded 100%, ranging from 100% to 900%, aligning with ideal dressing characteristics. The results obtained from this study demonstrate that QAS-PU/FC nanofibers have the capacity to establish a moist environment and absorb excess wound exudate.

#### 3.1.6 Mechanical properties

Tensile strength and elongation at break are significant mechanical parameters that determine the performance of nanofiber-based dressings (Fernandes et al., 2022). In normal skin, the tensile strength is usually in the range of 2.516 MPa and about 70% elongation at break (Ma et al., 2017). The experimental results are displayed in Figures 2C–F. Following grafting with acrylic esters, the tensile strength of the fiber membrane increased by 0.8–0.89 MPa compared to pre-grafting (QP), while the elongation at break decreased from 124 ± 2.7% to 67.4 ± 4.6%. The findings indicate that grafting acrylic esters enhances the balance between mechanical strength and flexibility in nanofiber wound dressings.

#### 3.1.7 Thermogravimetric performances

Thermal analysis can be used to determine the heat flux and heat loss characteristics of materials (Ijaola et al., 2023). As shown in Figure 2H, all three fiber membranes exhibit endothermic peaks corresponding to the melting point of PCL (approximately 65 °C). A broad peak around 400 °C indicates significant endothermic reactions, signifying the decomposition and disintegration of the PCL structure.

As demonstrated in Figure 2G, the findings suggest that QAS-PU/FC displays reduced decomposition rates and enhanced thermal stability, with a weight loss of approximately 30 wt.% observed between 55 °C and 350 °C. In the second stage of the experiment (QAS-PU/FC, QAS-PU, and FC), a rapid decomposition of the materials was observed at the designated temperatures. This resulted in a substantial weight reduction of approximately 70% of the initial weight. At a temperature of 600 °C, the weight loss was approximately 80 wt.%. The Janus nanofiber dressing QAS-PU/FC exhibits higher stability at temperatures above 100 °C, rendering it a promising material for applications.

### 3.2 Cell compatibility of nanofibers

**FIGURE 3:**
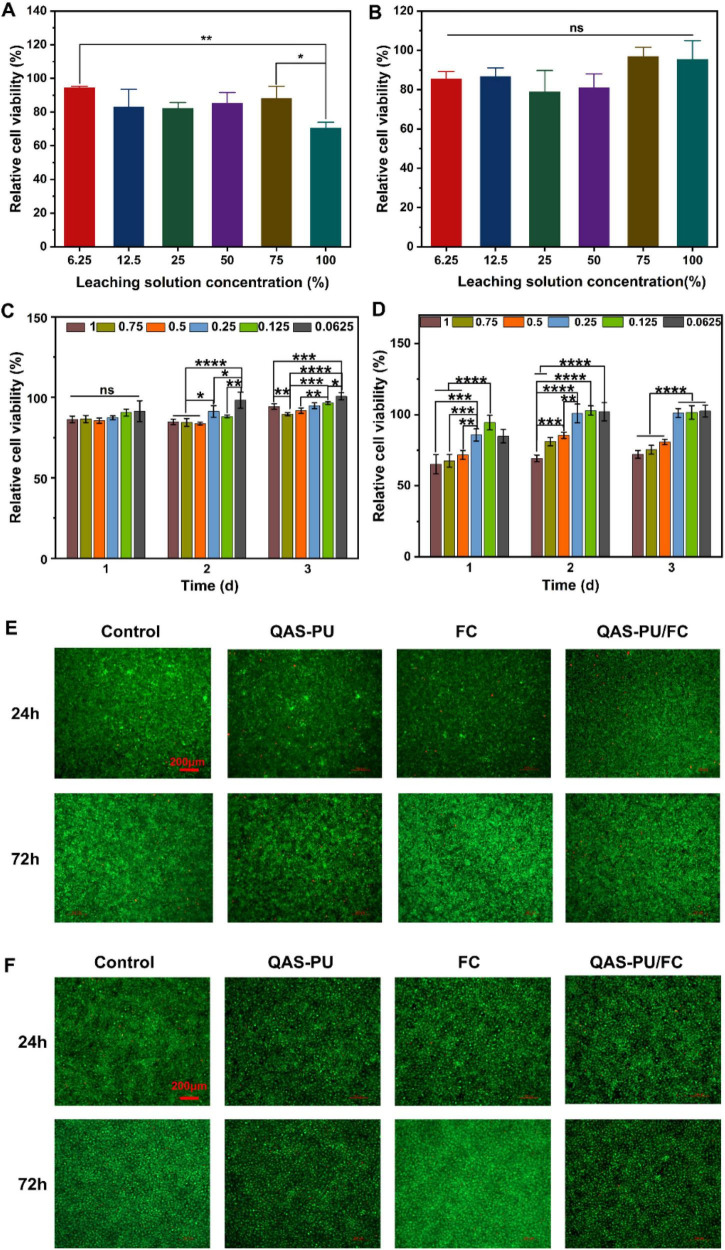
**(A)** Cell viabilities of L929 fibroblast cells (*n* = 4). **(B)** Cell viabilities of NIH-3T3 fibroblast cells (*n* = 4). **(C)** Relative proliferation rate of NIH-3T3 cells (*n* = 4). **(D)** Relative proliferation rate of L929 cells (*n* = 4). **(E)** L929 cell live-dead staining assay. **(F)** NIH-3T3 cell live-dead staining assay. *p < 0.05, **p < 0.01, ***p < 0.001, ****p < 0.0001 and ns means insignificant.

Good biocompatibility is a prerequisite for successful *in vivo* treatment (Zhang et al., 2023). The experimental findings suggest that the 24-h survival rate of NIH-3T3 cells on the dressing was greater than 75%, indicating that the QAS-PU/FC dual-layer fiber membrane dressing does not induce toxic side effects on the growth and metabolism of NIH-3T3 cells. However, while the dressing extract exhibited concentration-dependent inhibitory effects on L929 cells, its relative cell viability at the highest concentration (100%) remained at approximately 70%. There was a significant difference in cell viability compared with the 75% extract group; when the concentration was lower than 50%, the cell viability remained above 80%, showing no obvious cytotoxicity. These results further demonstrate that QAS-PU/FC is suitable for use as an intended wound dressing (Figures 3A, B).

As demonstrated in Figures 3C, D, following a 3-day co-culture of NIH-3T3 cells with the extract, no inhibition of cell growth was observed. Conversely, after 1 day, for L929 cells, when the extract concentration exceeded 25%, the relative growth rate fell below 75%, with toxicity classified as Grade 2 or 3. The initial mild toxicity at high extract concentrations can be attributed to the release of quaternary ammonium salts (QAS), whose positive charges may temporarily interfere with cell membranes. As the incubation time prolonged, collagen fully exerted excellent biocompatibility and effectively promoted cell adhesion and proliferation. These results indicated that the QAS-PU/FC composite dressing possessed qualified biocompatibility within an appropriate concentration range.

L929 cells and NIH-3T3 cells were subjected to staining using a live/dead staining solution. As demonstrated in Figures 3E, F, the cells exhibited uniform adhesion to the culture dishes, displaying a spindle-shaped morphology with a smooth surface devoid of wrinkles, analogous to the negative control group. The cells in the dressing group exhibited vigorous proliferation and growth, suggesting that the QAS-PU/FC double-layer membrane effectively promotes cell proliferation. Moreover, the green fluorescence intensity in the FC group was significantly higher than in the other groups, which can be attributed to collagen’s role in promoting cell proliferation and counteracting the slight inhibitory effect of QAS.

**FIGURE 4:**
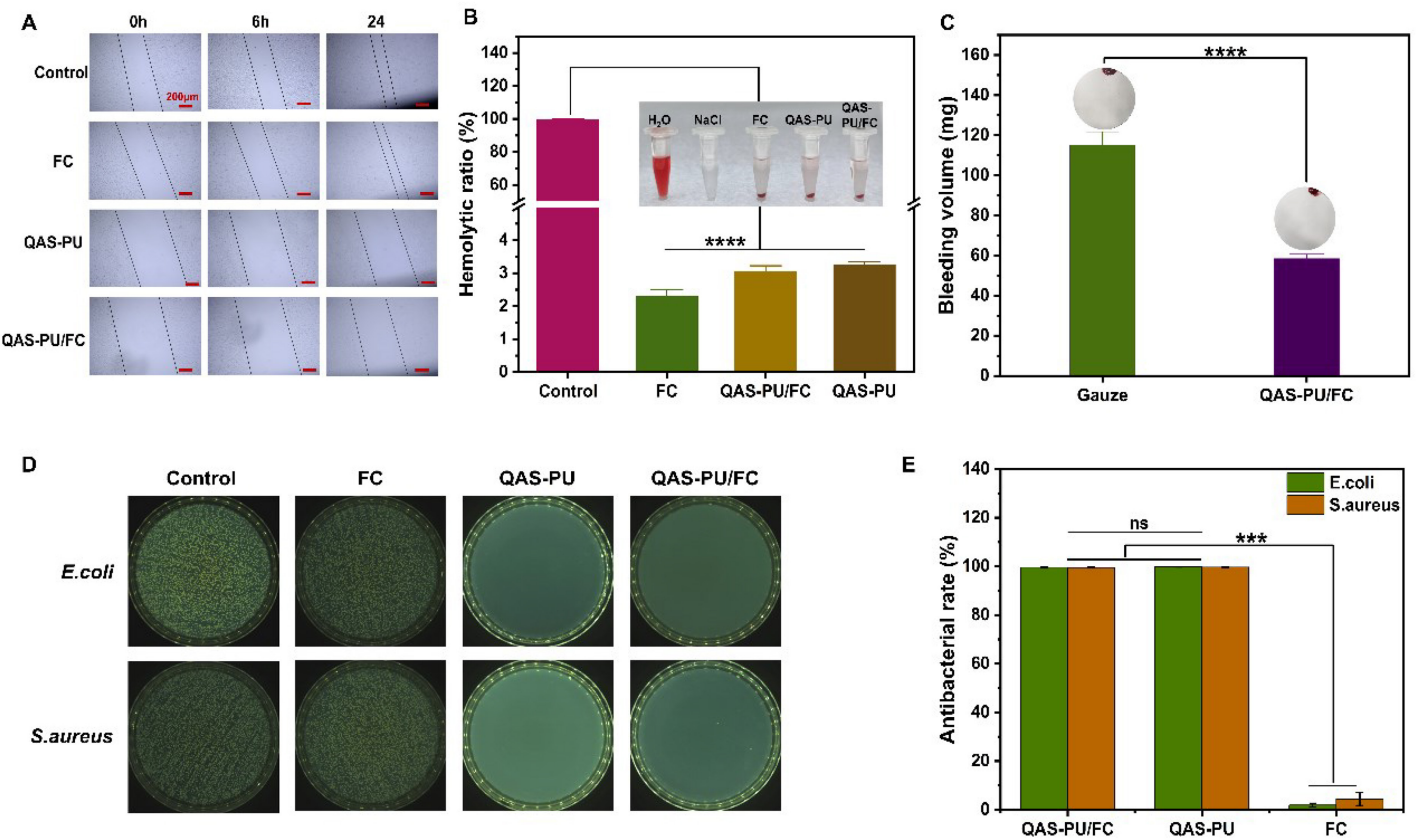
**(A)** Cell migration assay after cultured for 0, 6, and 24 h. **(B)** Statistical hemolysis ratio of NaCl, H_2_O, FC, QAS-PU/FC, and QAS-PU nanofiber, *n* = 3 (insert: hemolytic photographs). **(C)** Statistics of blood loss without treatment or with nanofiber treatments, *n* = 3 (insert: schematic diagram of the liver incision model). **(D)** The counter board pictures of *E. coli* and *S. aureus* growth on QAS-PU/FC, QAS-PU, and FC nanofibers (*n* = 3). **(E)** Antibacterial rates of QAS-PU/FC, QAS-PU and FC Nanofibers; ***p < 0.001, ****p < 0.0001 and ns means insignificant.

As outlined in the described methods (Li R. et al., 2025), The creation of uniform scratches on a monolayer is the objective of this experiment. Photographs were taken at 0, 6, and 24 h post-scratch formation (Figure 4A). At 0 h, scratches were clearly visible with well-defined edges across all groups. Following a period of 6 h, the edge cells began to migrate toward the area of the scratch. The cells in the control group exhibited the fastest migration rate, while QAS-PU-treated cells migrated slowly, an effect that is likely attributable to the toxic effects of quaternary ammonium salts. Furthermore, the migration rate of the QAS-PU/FC group was found to be marginally lower than that of the control group. Following a 24-h period, the cells from the control group had almost entirely covered the scratch area. Conversely, the FC group cells demonstrated a more pronounced capacity for scratch closure, a phenomenon attributed to collagen’s role in facilitating cell migration. The present study posits that the QAS-PU/FC composite fosters cell growth and exhibits augmented migratory capacity.

### 3.3 Hemocompatibility and hemostatic efficiency

The results of the hemolysis rate test are displayed in the Figure 4B. In the positive control group, red blood cells underwent an expansion in volume subsequent to water absorption, resulting in the formation of a clear red liquid. All other groups exhibited varying degrees of red blood cell sedimentation, indicating differences in hemolysis rates among the dressings. Specifically, the hemolysis rates for FC dressing, QAS-PU/FC dressing, and QAS-PU dressing were 2.32 ± 0.19%, 3.06 ± 0.16%, and 3.27 ± 0.07%, respectively all below the 5% threshold specified in ISO 10993-4 standards (Xie et al., 2019; Li Q. et al., 2025). Consequently, these dressings did not induce hemolytic reactions.

The hemostatic performance of the blank dressing QAS-PU/FC was evaluated using a mouse liver hemorrhage model (Figure 4C). The study showed that the mean volume of the QAS-PU/FC group (58.3 ± 2.6 mg) was significantly lower than that of the blank group (115 ± 7 mg), suggesting that the material has a higher hemostatic efficacy *in vivo*. This may be due to the introduction of a positive charge by the grafted quaternary ammonium salt, which activates the aggregation of negatively charged platelets thereby promoting blood coagulation and forming a physical hemostatic barrier through a dense fibrous structure.

### 3.4 Antibacterial properties

As demonstrated in Figures 4D, E, the QAS-PU and QAS-PU/FC membranes exhibited excellent antimicrobial performance due to their quaternary ammonium salt antimicrobial components having almost 100% antimicrobial efficiency by attracting and negatively charging bacterial cell membranes through positive charge. Conversely, the FC membrane exhibited no substantial antibacterial activity, with its colony counts demonstrating marked similarity to the control group. Despite the fact that QAS-PU/FC is a composite Janus nanofiber dressing formed by FC and QAS-PU, its antibacterial efficacy is comparable to that of the monolayer QAS-PU.

### 3.5 Wound healing promoting efficacy *in vivo*

**FIGURE 5:**
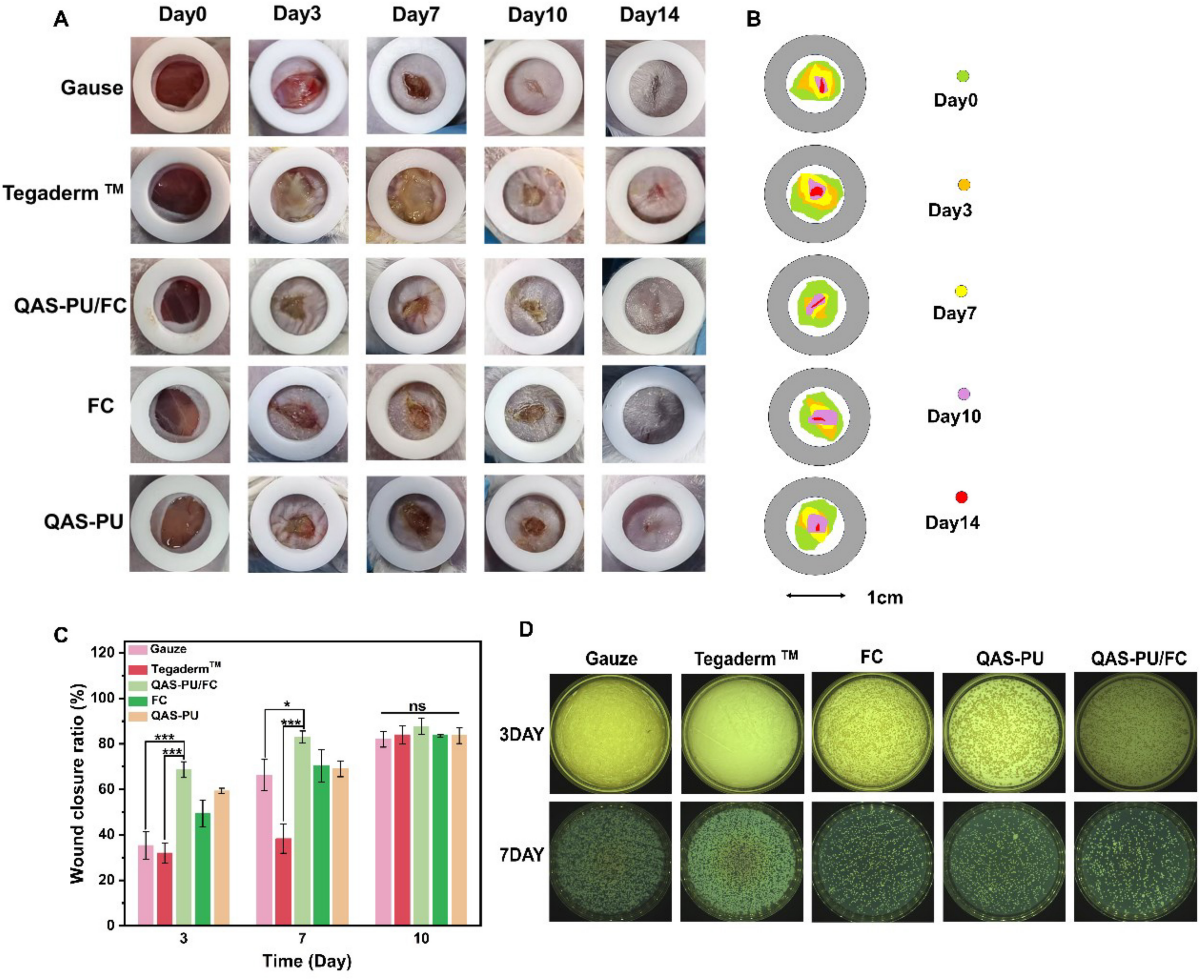
**(A)** Pictures of bacterial-infected wounds in mice in each group on 0, 3, 7, 10, and 14 days. **(B)** Diagram of healing within 14 days. **(C)** Wound healing rate of infected bacteria wounds in mice on days 3, 7, and 10. **(D)** Resistance of mouse tissues to infection at 3 and 7 days of inoculation with *S. aureus*. *p < 0.05, ***p < 0.001 and ns means insignificant.

As illustrated in Figures 5A, B, photographic documentation of the healing process of five different wound dressings was conducted at 0, 3, 7, 10, and 14 days after the application of each dressing to the wound bed. As demonstrated in Figure 5C, the rate of wound healing in mice at the 14-day stage is illustrated.

Twenty-four hours after wound infection, all groups exhibited signs of suppuration and bacterial infection. Following a period of 3 days, the QAS-PU/FC and QAS-PU groups exhibited significantly superior anti-infective effects in comparison to the other groups. At the 3-day mark, the wound healing rates were 31 ± 14%, 59 ± 1.3%, and 49 ± 6.7% for the groups treated with sterile gauze, QAS-PU, and FC, respectively. It is noteworthy that the QAS-PU/FC group demonstrated a healing rate of 68 ± 3.3%, which was significantly higher than both the Gauze group (***p < 0.001) and the Tegaderm*™* group (***p < 0.001), accompanied by the most significant marginal contraction. After 7 days, the QAS-PU/FC group exhibited the highest wound healing rate at approximately 83 ± 3%, maintaining significant advantages compared with the gauze group (*p < 0.05) and the Tegaderm™ group (***p < 0.001); the latter showed the lowest healing rate at roughly 38 ± 7%. By the 10th day, no signs of pus were observed on the wound surface. The QAS-PU/FC group demonstrated a healing rate of 87 ± 3.6%, which was marginally higher than the FC group (83 ± 0.3%) and the QAS-PU group (83 ± 3.3%). By day 14, the skin wounds in the experimental groups had largely healed, whereas the wounds in the control group remained incompletely closed. This discrepancy thus demonstrates the superior efficacy of QAS-PU/FC in promoting wound healing.

As demonstrated in Figure 5D, this graph illustrates the antibacterial efficacy against *S. aureus* on days 3 and 7 following the application of the dressing. After a period of 3 days, neither the gauze group nor the commercial dressing group exhibited antibacterial effects; however, the experimental QAS-PU group demonstrated outstanding antimicrobial activity. By the seventh day, a significant decrease in bacterial counts was observed, with the QAS-PU/FC and QAS-PU groups demonstrating superior antibacterial efficacy in comparison to the other groups. This phenomenon can be attributed to the antimicrobial action of quaternary ammonium salts.

**FIGURE 6:**
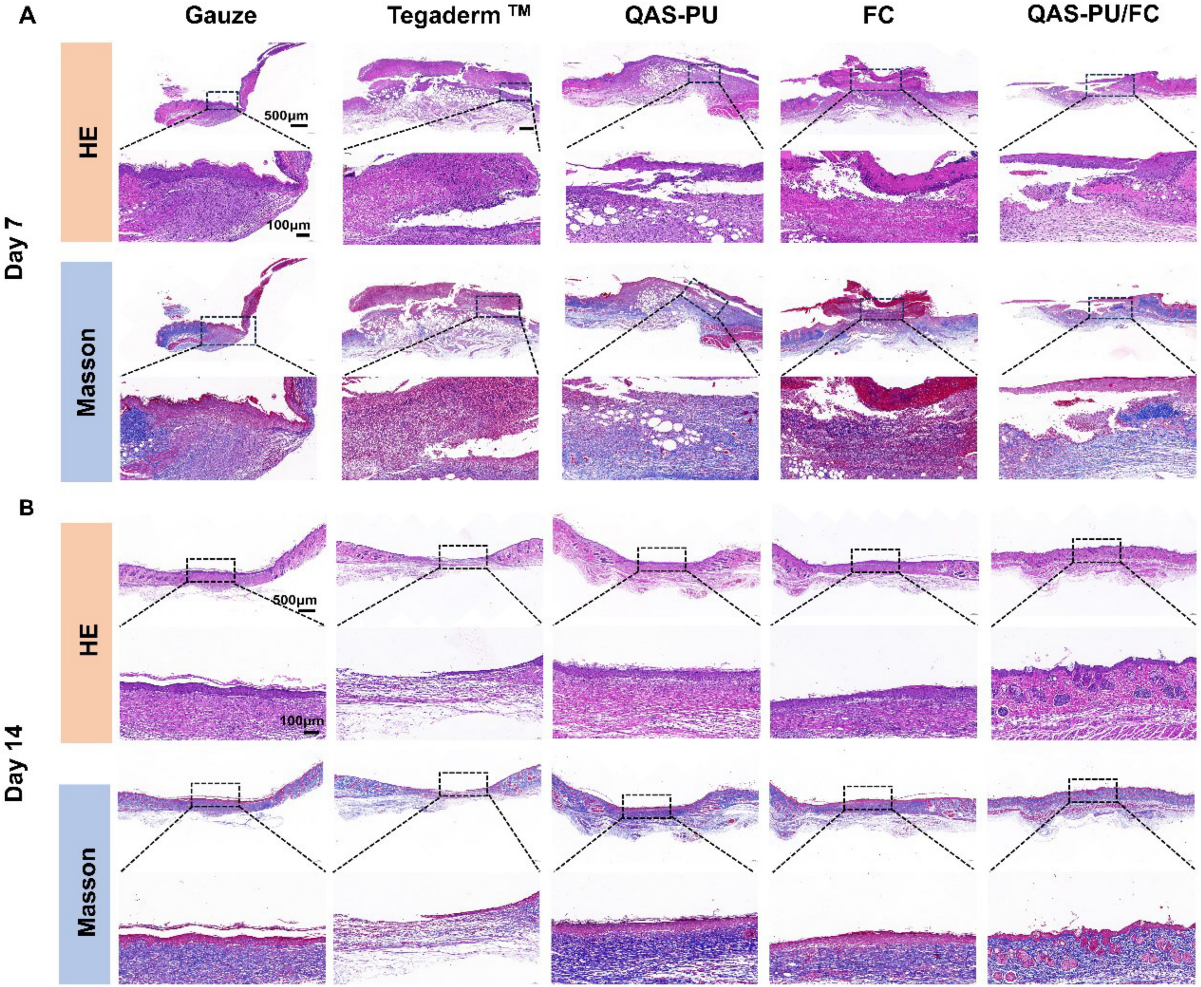
**(A)** HE and Masson staining images of wound tissue on day 7. **(B)** HE and Masson staining images of wound tissue on day 14.

Key indicators of the wound healing process include skin remodeling, fibroblast proliferation, and granulation tissue formation. H&E and Masson staining aid in assessing tissue regeneration at the wound site. Tissue samples were collected on days 7 and 14 for sectioning and staining. H&E and Masson staining results are shown in Figures 6A, B. On day 7, the gauze group, Tegaderm TM group, and FC group showed significant inflammatory cell infiltration, while the QAS-PU/FC group and QAS-PU group exhibited minimal inflammatory cell infiltration. By day 14, the Tegaderm TM group showed no signs of wound regeneration. In contrast, other groups exhibited varying degrees of regeneration, including vascular, epidermal, dermal, and skin appendage regeneration. Compared to other groups, tissue repaired and treated with QAS-PU/FC appeared more intact. This phenomenon can be attributed to the synergistic effect of collagen and quaternary ammonium salts in accelerating wound healing.

The staining results demonstrated that the blue coloration of the wound specimens from the QAS-PU/FC group and FC group was more pronounced than that of the control group and other groups at the 7th day of the initial healing stage of the wounds. This finding indicated that the collagen deposition in the QAS-PU/FC group was more evident than in the other groups. The QAS-PU group and the FC group demonstrated greater intensity and significance than the gauze group and the commercially available dressing. The tissues provided mechanical support, thereby promoting cellular proliferation and differentiation, which in turn is conducive to the generation of granulation tissue. On the 14th day, an increase in the number of neonatal hair follicles was observed in the QAS-PU/FC group. This resulted in collagen fibers that were thin and long, and exhibited an orderly distribution. In contrast, the other groups exhibited thick, irregularly arranged collagen fiber rows.

**FIGURE 7:**
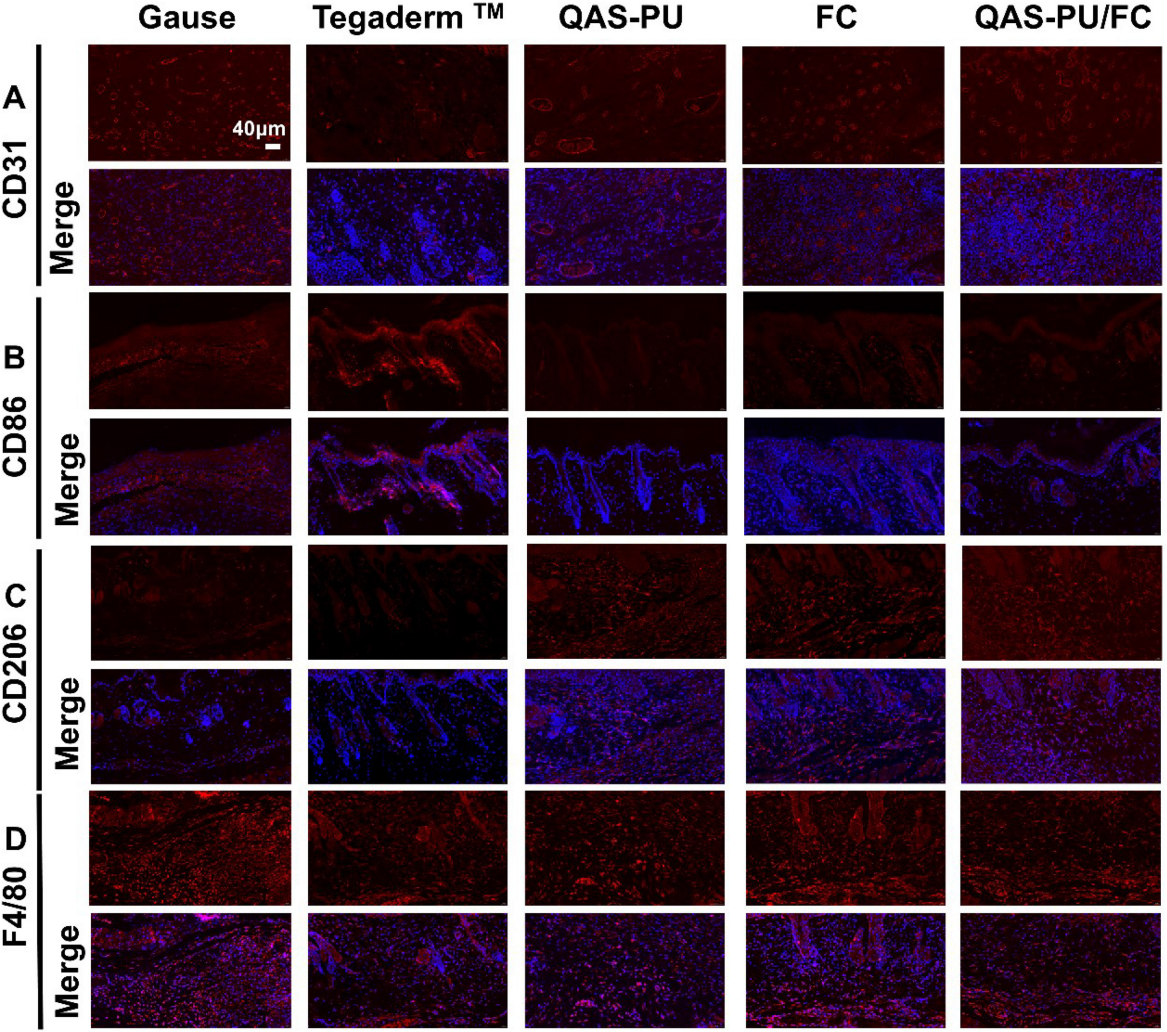
**(A)** CD31 immunofluorescence staining. **(B)** CD86 immunofluorescence staining. **(C)** CD206 immunofluorescence staining. **(D)** F4/80 immunofluorescence staining. Scale is 200 μm.

The process of wound healing is characterized by dynamic changes in macrophages and angiogenesis. Cytokines and marker proteins are frequently utilized in the detection of alterations within the wound microenvironment. F4/80 molecules, predominantly expressed on the surface of macrophages, are commonly employed as markers of mature macrophages. As illustrated in the Figures 7A–D, on the seventh day, the QAS-PU exhibited the strongest fluorescence intensity and the highest expression levels, as well as the most abundant number of macrophages. CD31 is a vascular endothelial marker. In comparison with the control group, the FC group and the QAS-PU/FC group exhibited enhanced fluorescence intensity and robust expression. This finding is indicative of neovascularization in regenerated tissues. The data suggest that these two dressings can significantly promote vascular regeneration of skin tissues and accelerate wound healing. In comparison with other groups, the Tegaderm™ group demonstrated the highest expression of CD86, indicating that the number of M2-type macrophages was superior in this group. In contrast to the Tegaderm™ group, the Gauze group, QAS-PU/FC group and FC group exhibited CD206 expression. The QAS-PU/FC group exhibited the highest relative expression, suggesting that M2-type macrophages constituted the predominant population in the QAS-PU/FC group.

The efficacy of the nanofiber composite membrane QAS-PU/FC in shortening the inflammatory period was evaluated by immunohistochemistry.IL-10 is an anti-inflammatory factor, and TNF-α is a characteristic pro-inflammatory cytokine that is closely associated with inflammatory responses during the early healing period. As demonstrated in the Supplementary Figures 1A, B, the image depicts a week of mouse tissue. The 7-day period is considered to be the midpoint of the natural healing phase. As demonstrated in the figure, the tissue specimens on the seventh day were subjected to immunohistochemical TNF-α staining experiments, which indicated that the expression of TNF-α was significantly lower in the FC group, the QAS-PU group, and the QAS-PU/FC group compared with the Tegaderm™ group. This suggests that the level of inflammation was higher in the commercially available dressing group compared with the other groups. The expression of IL-10 was significantly higher in the QAS-PU/FC group. The transition from the hemostatic/inflammatory stage to the proliferative stage was accelerated in the QAS-PU/FC group, while the control group remained in the chronic inflammatory stage. The results indicated that the QAS-PU/FC nanofiber composite membrane could shorten the inflammatory phase and wound healing time.

## 4 Conclusion

In this study, the QAS-PU/FC nanofiber membrane demonstrated considerable potential in accelerating the healing of infected wounds. The QAS-PU/FC nanofiber membrane exhibited efficient antimicrobial properties, high water absorption capacity, good biocompatibility, and highly effective hemostatic properties. The synergistic effect of antimicrobial quaternary ammonium polyurethane in the surface layer and collagen in the inner layer promoted the reconstruction of skin matrix resulted in good wound repair and significant antimicrobial effect in mice. The results of *in vitro* experiments demonstrated that QAS-PU/FC could significantly promote cell migration and angiogenesis for wound healing. Moreover, the QAS-PU/FC nanofiber membrane has been demonstrated to regulate the expression of pro-inflammatory cytokines, such as IL-10, and to reduce the release of the inflammatory mediator, TNF-α. The results of the study on the repair of infected full-thickness skin wounds in mice demonstrated that QAS-PU/FC induced orderly collagen deposition, promoted cell proliferation, and significantly accelerated the healing of infected wounds. In conclusion, the developed QAS-PU/FC dressing was a multifunctional therapy for infected wound management and can be considered an ideal candidate for infected wound dressing.

## Data Availability

The datasets presented in this study can be found in online repositories. The names of the repository/repositories and accession number(s) can be found in the article/Supplementary material.
