# Supplementary material for: Bioinspired electrospun janus nanofibrous dressing for synergistic antibacterial activity and tissue regeneration
Source: Front Microbiol. 2026 Apr 10;17:1788110. doi: 10.3389/fmicb.2026.1788110 (PMC13106119; doi:10.3389/fmicb.2026.1788110)
Supplement: Supplementary file 1 [file Data_Sheet_1.docx]

**Preparation and Research of Electrospun Antibacterial Polyurethane/Collagen Composite Membranes**

**1. Preparation of QAS-PU/FC nanofibrous membranes**

The prepared FC electrospinning solution was injected into a 10 mL plastic syringe equipped with a standard, blunt-tip needle (21G, diameter: 0.6 mm) for spinning. Spinning parameters were set as follows: voltage 10 kV, spinning distance 10 cm, injection rate 1.2 mL/h, temperature 25°C, humidity 35 ± 5%, and take-up roller speed 300 rpm. The spinning parameters for QAS-PU were set as follows: voltage 8 kV, spinning distance 10 cm, feed rate 1 mL/h, with temperature, humidity, and drum speed as above. The QAS-PU/FC fiber membrane was obtained at the end of spinning. The composite membrane was placed in a vacuum drying oven and kept at room temperature for 24 h before further characterization and evaluation.

**2. Characterization of nanofibrous membranes**

The surface morphology of the fibers was analyzed using a scanning electron microscope. Before imaging, the samples were sputter-plated for 60 seconds in an argon atmosphere to make them conductive. Approximately 50 fibers were measured in the SEM images using Image J software, and the average fiber diameter was calculated for each sample to make a diameter distribution plot for each sample.

**3. Water vapor permeability**

9 mL of PBS buffer was added to a 10 mL EP tube and weighed. The weight of the experimental group was recorded as W_0_ and the weight of the control group was recorded as W_1_. Each sample was fixed over the mouthpiece of the centrifuge tube. The tubes were placed in an oven at 37°C for 24 h, the samples were removed and weighed again. The weight of the experimental group was recorded as W_2_ and the weight of the control group was recorded as W_3_. The control group was not covered with the dressing. The experiment was repeated three times and the results were averaged. WVTR was calculated using the following formula:

$$WVTR\left( \% \right)=\frac{W_{0}-W_{2}}{W_{1}-W_{3}}\times100\%$$

**4 Water absorption capacity**

To estimate the ability of wound dressing to absorb wound exudate, the absorption capacity was calculated by the following method. Firstly, the dry dressing was weighed as W_0_ and then soaked in PBS solution until dissolution equilibrium, then the dressing was gently transferred to a petri dish and left for 1 minute to remove excess liquid from the surface. The weight of the dressing was then weighed as W_1_. The water absorption capacity was calculated from the following formula and the experiment was repeated three times.

$$Water Uptake Rate\left( \% \right)=\frac{W_{1}-W_{0}}{W_{0}}\times100\%$$

**5 Water contact angle of wound dressings QAS-PU/FC, FC, QAS-PU**

The water contact angle (WCA) of the wound dressing QAS-PU/FC was determined on a contact angle analyser. A droplet of water (approximately 10 μL) was placed on the fiber surface and the WCA was recorded for one minute.

**6 Tensile property**

Through the electronic universal test tensile machine, in accordance with the standard test method ASTM D-638, at room temperature and suitable humidity for all tests. A standard dumbbell-type specimen of size 20 mm × 2 mm was used. The experiment was repeated three times, and the tensile strength at break and elongation at break are calculated respectively, and the results are given as average values.

**7 Thermal behavior**

The thermal stability of the electrospun nanofiber dressings was evaluated using a thermogravimetric and simultaneous thermal analyzer. Weigh 7 ±2 mg of dry sample in an imported aluminum pan with a one-in-a-million balance, enter the weight, purge the Differential Thermal Scanning Calorimetry Analyzer with high purity nitrogen gas, set the rate of temperature increase to 10 K/min, set the test temperature to 25~800°C, and collect the curves.

**8. Cytotoxicity assay (CCK-8) test for QAS-PU/FC**

Fibrous membranes were sterilised by UV light for 30 min on each side for CCK-8 testing. Cytotoxicity magnitude of bilayer fibrous membranes was measured using L929 from mouse fibroblasts and NIH-3T3 from mouse embryonic fibroblasts. Cells were cultured in a carbon dioxide incubator at 37°C with 5% CO_2_. A cell suspension containing 5 × 10^4^ cells/mL of L929 and NIH-3T3 cells was prepared and added to the sample wells of a 96-well plate at a volume of 100 µL per well. Phosphate-buffered saline (PBS) was added to the blank control wells and the plate was incubated for 24 h. After this time, the medium was removed and the original sample extract and its dilutions were added to the plate. The high sugar medium was selected as the blank control and four replicate wells were set up for each concentration. The cells were then incubated under the extract for a further 24 h. The 96-well plate was then removed, the liquid aspirated, and 100 µL of test solution (10-fold dilution of CCK-8) added in the dark. The absorbance value of each well was detected by enzyme immunoassay at 450 nm. Cell viability was calculated using the following formula:OD

$$Cell viability(\%)=\frac{{OD}_{sample}-{OD}_{CCK-8}}{{OD}_{control}-{OD}_{CCK-8}}\times100\%$$

where OD_sample_ is the absorbance value of the experimental group, OD_control_ is the absorbance value of the blank group, and OD_cck-8_ is the absorbance value of CCK-8.

**9. Cell proliferation assay**

Firstly, 100 μL of the cell suspension was added to each well of a 96-well plate and 5,000 cells were added to each well. Four replicate wells were set up for each experimental group and a ring of phosphate-buffered saline (PBS) was added around the perimeter of the plate to minimize evaporation effects. The plates were placed in an incubator at 37 °C and 5% CO₂, and the medium was aspirated after 24 h to allow the cells to attach to the wall. The experimental group was given a medium containing cell extract and its dilution, while the blank group was given a medium without extract. The medium was aspirated after incubation in the incubator for 24, 48 and 72 h. Avoid exposing the medium to light, then add 100 μL of assay solution (10-fold dilution of CCK-8) to each well. After incubating for 1 h, detect the absorbance value at 450 nm for each well using ELISA.

**10. Cell Scratch wound assay**

L929 cells were cultured in 12-well culture plates at an initial inoculum density of 0.2 × 10^6^ cells/mL/well. After incubation for 24 h until the bottom was covered by L929 cells, the bottom of the cell-well plate was scraped with a 200 μL pipette tip to produce linear uniform scratches. The medium was later replaced by adding the original concentration of QAS-PU/FC/, QAS-PU, and FC three-material leachate with PBS to remove the dislodged cells. The cells were observed and photographed by fluorescence microscope at 0, 6, and 24 h, respectively. NIH Image J software (USA) was used to measure the wound area. The percentage coverage of the wound area relative to the initial wound area (0 h) at each time point was tested. The test was repeated three times for each group.

**11. *In vitro* antibacterial assay**

The materials were completely submerged in culture medium and incubated with *E. coli* and *S. aureus* for 24 h. The culture solution was then diluted 1000 times and 100 μL of it was added to Luria-Bertani (LB) medium and spread well using a metal ring. The bacteria were incubated at 37°C for 24 h and then photographed. The bacterial solution not co-cultured with the material was considered as control. Three trials were performed under the same conditions. The rate of bacterial reduction was calculated by counting the number of CFUs on the agar plates using the following formula:

$$Reduction rate of bacteria \left( \% \right)=\frac{q_{control}-q_{sample}}{q_{control}}\times100\%$$

Where q_control_ is the number of colonies in the control group and q_sample_ is the number of colonies in the experimental group

**12. *In vitro* haemostasis test**

The liver was removed via an abdominal incision and the secretion was cleaned up with filter paper. A 20G needle was then used to induce bleeding to form a cylindrical wound with a diameter of approximately 1 mm. The sample dressing was quickly applied to the wound site and the gauze-treated group was used as the control group. The amount of blood loss was measured after 2 min. Blood spillage was observed using a pad of filter paper, and the weight of the filter paper that had absorbed the blood was measured to assess the haemostatic performance of the prepared materials. The weight of the filter paper absorbing blood was measured to assess the haemostatic performance of the prepared materials.

**13. Haemolytic properties test**

Balb/c male mice (25-28 g, about 8 weeks old) eyeball blood 2 mL, divided into two anticoagulant tubes, blood centrifugation to remove the supernatant to separate the erythrocytes, and then add saline washing centrifugation several times, to be clarified supernatant, and then add 30 mL of saline resuspension dilution, to get the desired erythrocyte suspension.

To evaluate the hemocompatibility of the hydrogels, 1000 μL of erythrocyte suspension was added to the material and incubated at 37 °C for 1 h. Afterwards, the mixed solutions were centrifuged and 100 µL of each set of supernatant was aspirated and added to a 96-well microtiter plate. The OD values were read by an enzyme marker at 540 nm. 0.1% Triton x-100 was used as a positive control, while saline was used as a negative control. The hemolysis rate is calculated using the following formula:

$$Hemolysis rate(\%)=\frac{{OD}_{x}-{OD}_{p}}{{OD}_{t}-{OD}_{p}}\times100\%$$

Where *OD_x_* is the absorbance value of the experimental group; *OD_p_*, of the negative control group; and *OD_t_*, of the positive control group (0.1% Triton X-100).

**14. *In vivo* wound healing assay**

First, the mice were anesthetized by intraperitoneal injection of 5% chloral hydrate solution (100 μL/20 g). Then a full-layer wound (about 8 mm) was prepared on the back of the mice. Then, 10 μL of active *S. aureus* was taken and added to the wound.

All mice were treated 24 h after infection. Wound healing photographs were taken on days 0, 3, 7, 10, and 14 post-treatment and the wound area was analyzed and quantified using Image J. *A_0_* is the initial wound area (0d) and *A_t_* is the post-treatment wound area (3d, 7d, 10d, 14d, and 21d). The Wound closure ratio was calculated as follows:

$$Wound closure ratio(\%)=\frac{A_{0}-A_{t}}{A_{0}}\times100\%$$

Two mice in each group were executed on the 7th and 14th day after mouse modeling, and the specimens were extracted from the wounds, the samples were rinsed with saline, and the specimens were fixed in 4% paraformaldehyde tissue fixative for use. Tissue samples were paraffin-embedded and longitudinally cut, and H&E and Masson stains were used to observe inflammatory cell infiltration, fibroblast proliferation, and collagen deposition, and immunohistochemical staining for IL-10 and TNF-α factors was performed on skin tissue sections on the seventh day to evaluate inflammation during wound healing.

**15. The present study investigates the use of immunofluorescence staining for the detection of IL-10 and TNF-α.**
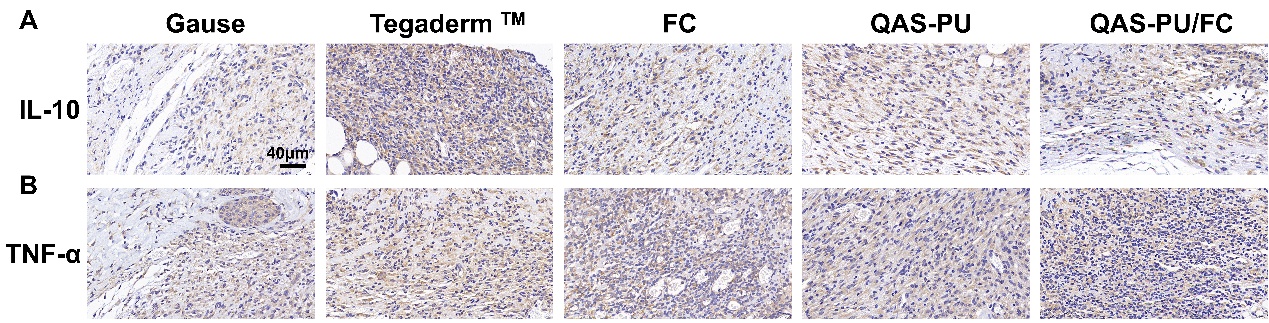


**Figure S1** (A) IL-10 immunohistochemical staining; (B) TNF-α immunohistochemical staining.
